# Dental Implants Used for Orthodontic Anchorage in Patients with Treated Stage IV Periodontitis: A Retrospective Case–Control Study

**DOI:** 10.3390/jfb17010049

**Published:** 2026-01-18

**Authors:** Shing-Zeng Dung, I-Shiang Tzeng

**Affiliations:** 1 Department of Dentistry, Taipei Tzu Chi Hospital, Buddhist Tzu Chi Medical Foundation, No. 289, Jianguo Rd., Xindian Dist., New Taipei City 23142, Taiwan; 2 Department of Surgery, College of Medicine, Tzu Chi University, Hualien 970374, Taiwan; 3 College of Dentistry, National Yang Ming Chiao Tung University, Taipei 112304, Taiwan; 4 Institute of Oral Medicine and Materials, College of Medicine, Tzu Chi University, Hualien 970374, Taiwan; 5 Department of Research, Taipei Tzu Chi Hospital, Buddhist Tzu Chi Medical Foundation, No. 289, Jianguo Rd., Xindian Dist., New Taipei City 23142, Taiwan

**Keywords:** dental implants, orthodontic anchorage procedures, periodontitis

## Abstract

Little is known about the effects of orthodontic loading on dental implants used for orthodontic anchorage in patients with Stage IV periodontitis. This retrospective case–control study included 58 dental implants in 24 patients with treated Stage IV periodontitis. The dental implants were used for both chewing function and orthodontic anchorages. The outcome measures included peri-implant marginal bone loss and peri-implantitis. Pair *t*-test and Wilcoxon rank-sum test were used to analyze the impact of implants as orthodontic anchorage on marginal bone loss (MBL) and peri-implantitis. No implants were lost during the 17-year follow-up. There was no statistically significant difference in the MBL and incidence of peri-implantitis between implants used as orthodontic anchorage and non-anchorage controls. (*p* > 0.05). Poor oral hygiene (*p* = 0.05), one-piece implants (*p* = 0.05) and implants with a keratinized mucosa < 2 mm (*p* = 0.015) were associated with a higher risk of peri-implantitis. Results from the present long-term study indicated that dental implants could be successfully used as orthodontic anchorage in periodontal compromised patients.

## 1. Introduction

In addition to the severity and complexity characteristics of stage III periodontitis, patients with Stage IV periodontitis often present with esthetic and functional issues, such as tooth elongation, tilting, flaring, diastema, and missing teeth, warranting interdisciplinary orthodontic treatment and oral rehabilitation [1]. Obtaining proper orthodontic anchorage is often challenging in these patients owing to partial edentulism and reduced periodontal support [2,3,4,5,6,7,8,9,10,11]. Two major types of implants used for orthodontic anchorage are mini-implants or mini-plates used exclusively for temporary anchorage devices and dental implants with a dual orthodontic–prosthetic function. There is a clinical knowledge gap in long-term clinical outcomes between implants used solely for prosthetic purposes and implants with a dual orthodontic–prosthetic function.

Osseointegrated titanium implants are widely used to replace missing teeth in patients with partial edentulism during oral reconstruction [12]. These prosthetic implants have also been proposed for orthodontic anchorage, particularly in cases in which the periodontally compromised or mutilated dentition lacks sufficient anchorage for effective tooth movement [2,3,4,5,6]. In such scenarios, dental implants may initially serve as orthodontic anchorage and subsequently function as prosthetic abutments, contributing to long-term stability, function, and comfort [6,7,8,9,10,11].

Experimental animal studies have shown that dental implants subjected to orthodontic forces maintain their stability and effectively address a wide range of orthodontic and prosthetic challenges [13,14,15,16,17]. Clinical use in humans has also revealed that dental implants as orthodontic anchorage can support various types of tooth movement [3,4,5]. Roberts et al. [18] used a two-stage endosseous implant in the retromolar region of the mandible to provide rigid anchorage, successfully transpositioning two molars 10–12 mm mesially into an atrophic edentulous ridge. In a prospective study conducted by Higuchi and Slack [19], titanium implants were placed in the posterior mandible of seven patients following the Brånemark protocol. These implants were used to either protract the entire dentition in the maxilla and mandible or retract and correct Class III anterior crossbite malocclusions in patients with missing molars. Except for one implant, all implants were placed in the retromolar areas and were not intended for prosthetic use.

Studies by Odman et al. [20], Kokich, and others [3] have demonstrated that dental implants can serve as reliable alternatives to orthodontic anchorage in partially edentulous patients. Stage IV Periodontitis, defined by the 2017 world workshop classification, is the most severe form of periodontal disease with less than 20 teeth, leading to chewing difficulty and requiring multidisciplinary treatment to restore health, function and esthetics [1]. Despite numerous reviews on the topic [2,3,4,5,6,7,8,9,10,11], information on patients with Stage IV periodontitis remains limited. In addition, studies directly comparing the marginal bone level (MBL) in dental implants used for orthodontic anchorage versus those used solely for prosthetic purposes are limited [21,22,23,24]. Research focusing on the long-term outcomes and risk factors of dual-purpose implants is scarce.

The purpose of this retrospective case–control study was to test the null hypothesis that there are no significant differences in the clinical or radiographic outcomes of dental implants used for orthodontic anchorage between patients with Stage IV periodontitis and a control non-anchorage implant group over a follow-up period exceeding 17 years, and to identify risk factors associated with the use of dental implants as orthodontic anchorage in patients with treated Stage IV periodontitis.

## 2. Materials and Methods

### 2.1. Inclusion and Exclusion Criteria

All medically healthy patients with Stage IV periodontitis who were referred for periodontal, implant, and interdisciplinary therapy to the Department of Dentistry, Taipei Tzu Chi Hospital, between 2007 and 2016, were enrolled in this long-term case–control study. Patients who had received at least one implant for orthodontic anchorage and at least one implant solely for prosthetic support were eligible for inclusion. From the beginning how 34 patients were initially assessed, 10 were excluded, and 24 were ultimately included in the study. Although patients with severe periodontitis in this study were enrolled between 2007 and 2016, the diagnosis and classification of periodontitis were updated and applied according to the 2017 World Workshop classification.

This study was approved by the Institutional Review Board of Taipei Tzu Chi Hospital (IRB No. 13-IRB 41). The study was conducted in accordance with the principles outlined in the Declaration of Helsinki and all participants provided written informed consent for both the treatment plan and surgical procedures. This study followed the Strengthening the Reporting of Observational Studies in Epidemiology (STROBE) guidelines, as outlined in the Supplementary File.

Exclusion criteria included a history of alcohol or drug abuse, uncontrolled diabetes (glycated hemoglobin [HbA1c] > 8.5%), heavy smoking (>10 cigarettes per day), or any systemic disease that could compromise periodontal or orthodontic therapy.

Treatment planning was patient-centered and based on individual health, function, and esthetic needs. Each patient provided written informed consent for the interdisciplinary treatment plan and associated surgical interventions. Periodontal therapy was performed according to evidence-based guideline on the interdisciplinary treatment of stage IV periodontitis [1]. Basic periodontal treatment, including patient education, oral hygiene instructions, and scaling and root planing, was performed to control periodontal infection. Periodontitis patients received first complete thorough periodontal treatment to achieve disease stability, defined by low levels of bleeding on probing (BOP), shallow probing depths (typically ≤4 mm), and absence of suppuration, before proceeding with implant placement [25].

### 2.2. Periodontal and Implant Surgical Procedures and Postsurgical Care

All surgeries were performed by one of the authors (SD), a clinician with over 20 years of experience in periodontal and implant surgeries (for detailed information, see Dung [26]).

Open flap debridement or regenerative periodontal surgeries were conducted to thoroughly debride diseased root surfaces or regenerate lost periodontal tissues. An implant bed was prepared to optimize primary implant stability. Horizontal ridge deficiencies or the lateral wall of the maxillary sinus were augmented using biphasic calcium phosphate bone grafts (MBCP™, Biomatlante, Vigneux-de-Bretagne, France) and covered with collagen membranes (Neomem^®^, Citagenix, Laval, QC, Canada or EZ Cure™, Biomatlante, France). The flap was coronally repositioned and secured using resorbable Vicryl sutures (Ethicon J&J, Taipei, Taiwan) to promote primary wound healing.

Postoperatively, the patients were instructed to continue Augmentin 1000 mg (SmithKline Beecham Limited, Worthing, UK) twice daily to prevent infection. In addition, they were advised to use a 0.12% chlorhexidine mouthwash (Beauteeth Co., Ltd., New Taipei City, Taiwan) twice daily for 2 weeks. Patients were instructed to avoid tooth brushing and chewing in the surgical area for 4 weeks. The sutures were removed 2 weeks postoperatively, and supragingival mechanical plaque control was performed. Follow-up wound care was provided 2, 4, 8, and 12 weeks postoperatively. Supportive periodontal and peri-implant care (SPC) was administered every 3 months after the orthodontic treatment completion.

### 2.3. Implant Provisional Restoration and Orthodontic Treatment

Implant provisional restorations were fabricated and loaded at the appropriate time after implant placement. Self-ligating orthodontic brackets (Damon Q, Ormco, Orange, CA, USA) were used, and Damon orthodontic arch wires (Cu-Ni-Ti, 0.014” round wire) were inserted. Active orthodontic treatment was initiated immediately after the delivery of the provisional implant restorations, with adjustments performed every 1–2 months. The archwire sequence was: 0.014 NiTi, 0.016 NiTi, 0.014 × 0.025 NiTi, 0.016 × 0.025 NiTi. The implants used as anchorage were submitted to sliding, compression and traction forces by means of power chain and open coil springs. Dental implants used for orthodontic anchorage were under forces ranging from 100 to 200 g. SPC was provided at each orthodontic visit.

**Figure 1:**
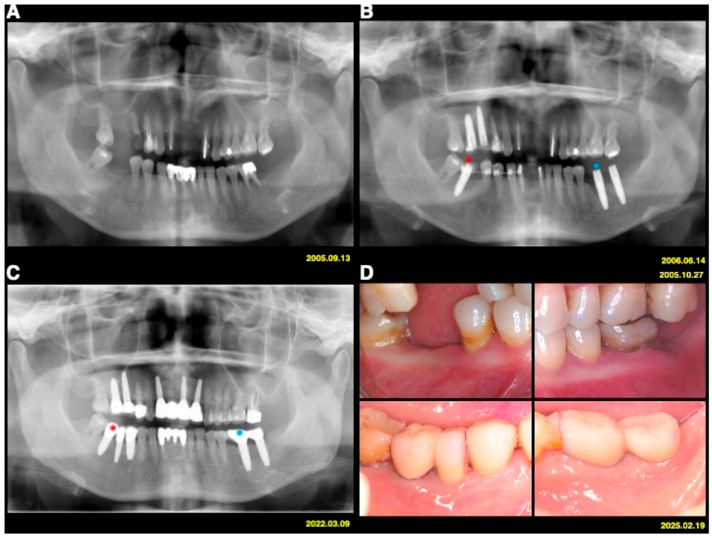
Case 1 (**A**) A 55-year-old female patient with Stage IV periodontitis. (**B**) Implants served as orthodontic anchorage (test; red stars) and for prosthetic rehabilitation only (controls; blue stars). (**C**) Radiographs in 2022 showed no periodontal or peri-implant bone loss. (**D**) Clinical findings at 20 years post-treatment demonstrated stable and healthy periodontal and peri-implant tissues.

**Figure 2:**
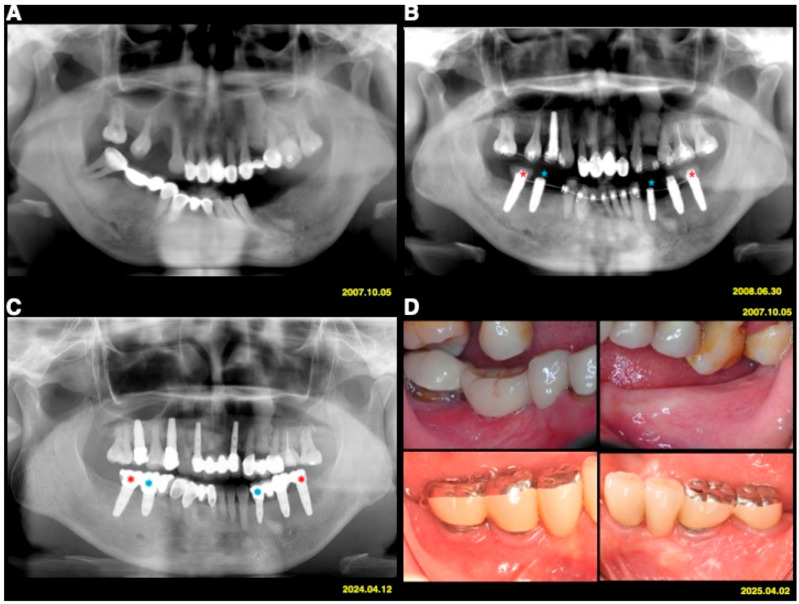
Case 2 (**A**) A 40-year-old female patient with Stage IV periodontitis. (**B**) Implants served as orthodontic anchorage (test; red stars) and for prosthetic rehabilitation only (controls; blue stars). (**C**) Radiographs in 2024 showed minimal periodontal and peri-implant bone loss. (**D**) Clinical findings at 17.5 years post-treatment demonstrated stable and healthy peri-implant tissues.

The test implants served as absolute orthodontic anchorages to facilitate molar uprighting, tooth retraction and realignment, or implant site development, whereas implants used exclusively for prosthetic purposes served as controls (Figure 1 and Figure 2). The use of implant anchorage significantly supported orthodontic treatment in patients with periodontally compromised dentition. A key advantage of this combined orthodontic–prosthetic approach was that the implants play a dual role, providing both anchorage and functional rehabilitation.

All the implants remained stable throughout the entire duration of orthodontic treatment. Orthodontic treatment period varied between 7 and 29 months. Upon completion of active orthodontic therapy, the appliances were removed and definitive cement-retained fixed implant prostheses were delivered. All the patients received fixed retainers to prevent relapses. SPC was continued every 3 months to maintain periodontal and peri-implant health.

### 2.4. Clinical and Radiographic Evaluation

Clinical examinations and standardized periapical and panoramic radiographs were obtained at the initial assessment, during orthodontic therapy, at the time of implant restoration, and at the final follow-up. The clinical parameters evaluated included the plaque score, BOP, probing depth, marginal tissue recession, and width of the keratinized mucosa (KM). Midfacial marginal tissue recession (MTR) was defined as the distance from the crown margin to the mucosal margin. The number of SPC per year was recorded.

**Figure 3:**
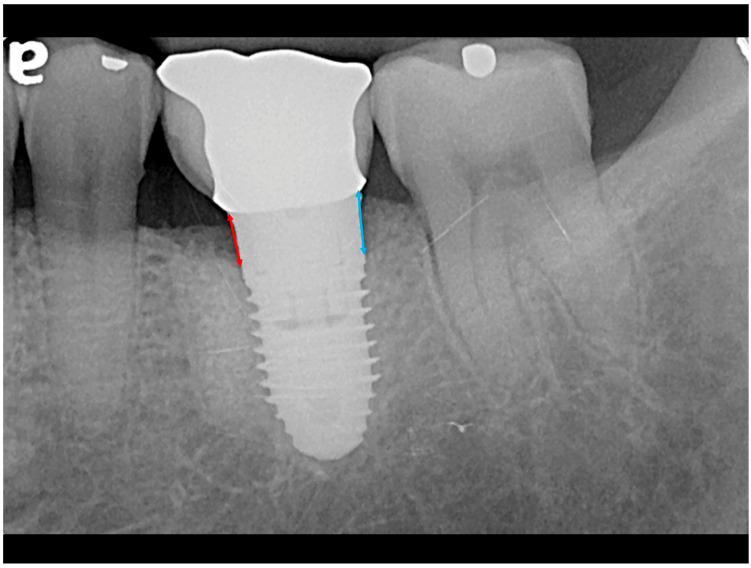
Measurement of marginal bone level. The marginal bone level was measured from the crown margin to the bone crest. Both the mesial (red line a or c) and distal (blue line, b or d) implant bone levels at crown delivery (a, b) and at follow-up (c, d) were measured. The mean mesial and distal bone levels were calculated as the average bone level for each implant. Average total bone loss was measured by deducting the baseline mean bone level (a + b/2) from the last follow-up bone level (c + d/2).

Baseline periapical radiographs were obtained at the initiation of orthodontic therapy with implants as reference for the MBL measurements. Radiographic assessments of the MBLs around the implants were conducted using digital imaging software (Infinitt Radiology PACS, ver 3.0.11.5_B9P3, Taipei, Taiwan). The length of the implant was used to adjusted the X-ray distortion. Each measurement was repeated three times by a single examiner at three different time points. Intra-examiner error is 0.06 mm. The MBL was measured from the crown margin to the alveolar bone crest (Figure 3). Both the mesial and distal bone levels were measured at the time of crown delivery (points a and b) and at the final follow-up (points c and d). The mean bone level for each implant was calculated as the mean of the mesial and distal measurements. The distortion ratio was used to adjust radiographic assessments for a coefficient derived from the true implant length/radiographic implant length ratio to account for radiographic distortion and magnification. Total bone loss was calculated by subtracting the baseline average bone level (a + b)/2 from the final follow-up average bone level (c + d)/2.

Following implantation and orthodontic therapy, the patients were recalled for clinical and radiographic evaluations at 1 month, 3 months, 6 months, and 1 year, and annually thereafter for up to 17 years. During each follow-up visit, SPC was performed and occlusion was reassessed. Implant survival and success were evaluated according to the established criteria [27,28,29].

### 2.5. Case Definitions of Peri-Implantitis

Case definitions of peri-implantitis were based on the consensus report of Workgroup 4 of the 2017 World Workshop on the Classification of Periodontal and Peri-implant Diseases and Conditions [30]. Peri-implantitis was defined as the presence of bleeding on probing and/or suppuration upon gentle probing, increased probing depth compared with previous examinations, and bone loss beyond the expected crestal bone-level changes associated with initial bone remodeling. In this long-term study, peri-implantitis was defined as MBL > 1 mm, accounting for a probing measurement error of approximately 0.8 mm and 0.5 mm bone level changes resulting from initial bone remodeling. The authors assumed that this is a relatively strict definition of peri-implantitis for a long-term study.

### 2.6. Risk Factors for MBL and Peri-Implantitis

Possible patient- and implant-related risk factors were evaluated, including oral hygiene (OH), compliance with SPC, timing of orthodontic loading, lack of KM (defined as KM < 2 mm), MTR, and duration of follow-up.

Compliance was assessed based on the average number of periodontal maintenance visits per year after surgery. Patients were categorized as highly compliant if they attended more than three visits per year, moderately compliant if they attended two visits per year, and poorly compliant if they attended only 0–1 visits per year. OH at the follow-up visits was evaluated using the O’Leary’s Plaque Score (PS) [31]. OH status was classified as good (PS < 20%), moderate (20% ≤ PS < 50%), or poor (PS ≥ 50%).

### 2.7. Statistical Analysis

Potential patient- and implant-related risk factors associated with MBL and peri-implantitis in implants used for orthodontic anchorage were analyzed. Pair t test and Wilcoxon rank-sum test were used to analyze the impact of implants as orthodontic anchorage and implant type on changes in MBL. Univariate analyses were performed using the Wilcoxon rank-sum test for continuous variables and chi-square test for categorical variables in more than two groups. All statistical analyses were conducted using R software (version 4.3.3), with a significance level set at 5% (α = 0.05).

## 3. Results

This study included clinical and radiographic evaluations of 58 implants placed in 24 patients, with a mean age of 49.03 ± 9.30 years. The mean follow-up period was 11.95 ± 3.68 years (range: 5–17 years). Table 1 summarizes the descriptive characteristics of the patient- and implant-related risk factors for implants used as orthodontic anchorage and non-anchorage controls. Among the study population, 27.6% demonstrated good OH, approximately 60.3% received SPC at least twice per year, and 74% of the patients had follow-up periods longer than 10 years. All implants were restored using fixed crown and bridge prostheses. Only five teeth from five patients were lost during the follow-up period. Two teeth were lost due to periodontitis, one due to dental caries, and two due to tooth fractures.

Of the 58 implants, 36 (62.1%) and 22 (37.9%) were two-piece bone-level cylindrical implants (Replace^®^ system, Yorba Linda, CA 92887, USA) and one-piece implants (Nobel Direct^®^, Yorba Linda, CA 92887, USA), respectively. Of the 29 implants utilized as orthodontic anchorage, one site (3.4%) was in the anterior region and 12 sites (41.4%) were in the maxilla. Seventeen implants (58.6%) were subjected to orthodontic loading within 3 months of placement. A total of 17.2% of the implants and 29.2% of the patients demonstrated an MBL greater than 1 mm. Notably, 51.7% of the implants had a KM width of less than 2 mm and 62.1% revealed no tissue recession with follow-up exceeding 10 years.

Table 2 presents the relationship between implant anchorage type and changes in MBL. The mean MBL was 0.43 ± 0.95 mm for anchorage implants and 0.28 ± 1.10 mm for non-anchorage control implants. Both the t-test and Wilcoxon signed-rank test indicated no statistically significant association between the MBL and orthodontic anchorage or implant type (*p* > 0.05).

Univariate analysis of patient- and implant-related risk factors for MBL is shown in Table 3. Among these factors, low compliance with the SPC was significantly associated with increased MBL (*p* = 0.004).

The baseline characteristics stratified by the presence of peri-implantitis are shown in Table 4. Of all the risk factors, OH and implant type demonstrated a moderate association with peri-implantitis, with borderline statistical significance (*p* = 0.05). Other variables, including compliance, timing of implant loading, KM < 2 mm, tissue recession, and duration of follow-up, were not significantly associated with peri-implantitis.

Table 5 summarizes the variables associated with peri-implantitis in implants used for orthodontic anchorage and in non-anchorage control implants. None of the evaluated patient- or implant-related factors showed a statistically significant difference in the incidence of peri-implantitis between the two groups (*p* > 0.05). Table 6 further evaluates the effect of orthodontic loading time on peri-implantitis. The results indicated that the timing of implant loading for orthodontic anchorage was not significantly associated with the development of peri-implantitis (*p* > 0.05).

## 4. Discussion

To the best of our knowledge, this is the first long-term case–control study to accept the null hypothesis that there is no significant difference in the MBL and peri-implantitis between dental implants used for orthodontic anchorage and those used solely for prosthetic purposes.

Specific studies directly comparing the MBL and survival rates of dental implants used for orthodontic anchorage versus those used solely for prosthetic support are limited [22,23,24]. In a 1- to 6-year case series involving 43 roughened-surface implants in 11 patients, Kato and Kato [22] also found that all implants used for orthodontic anchorage maintained osseointegration and continued to function effectively. There were no significant differences in the MBL or implant survival between implants used as orthodontic anchors and those used exclusively for prosthetics. Marta and Carlos [23] evaluated 93 implants used for orthodontic anchorage under forces ranging from 100 to 200 g in 38 partially edentulous patients. The results demonstrated that the implants could withstand orthodontic forces and maintain osseointegration without significant changes in the bone levels.

Marins et al. compared the MBL of osseointegrated implants subjected to orthodontic forces with that of implants used solely for prosthetic loading after 3 years of functional use [24]. Twenty-six implants were used for orthodontic anchorage, and 24 served as controls for prosthetic support only. These findings indicate that using implants for intraoral orthodontic anchorage does not compromise peri-implant tissue health or implant longevity. However, factors such as force magnitude and direction, implant location, and patient-specific characteristics (e.g., bone density and OH) may influence the outcomes. Additional high-quality, randomized, long-term studies are warranted to confirm these findings.

Several studies have suggested that in motivated and compliant patients with stably treated stage IV periodontitis, orthodontic tooth movement demonstrated no significant impact on the periodontal outcomes [32,33,34]. However, there is limited evidence regarding the treatment outcomes and risk factors of implants for orthodontic anchorage in patients with stage IV periodontitis. Univariate analysis of all patient- and implant-related risk factors in this study revealed that low compliance was the only factor significantly associated with a greater MBL (*p* = 0.004).

This long-term case–control study may be the first to evaluate the risk factors for peri-implantitis in prosthetic implants used for orthodontic anchorage. In the present study, only three patients were light smokers, and none developed peri-implantitis during follow-up. As a result, light smoking was not included as a risk factor in the statistical analysis. Among the identified risk factors, the use of one-piece implants and poor OH were moderately associated with the development of peri-implantitis. Given the borderline associations, small sample sizes, and wide confidence intervals, the findings should be interpreted with caution.

Previous studies have also shown that one-piece implants (e.g., NobelDirect), while eliminating the microgap, exhibit greater early MBL [35,36]. Immediate loading, multiunit construction, flapless surgery, cement-retained restorations, and other factors may also contribute to the observed bone loss in one-piece implants. The reason why one-piece implants used for orthodontic anchorage demonstrated a higher risk of peri-implantitis in this study remains unclear, warranting further investigation.

Berglundh et al. [37] reported that patients with a history of periodontitis, particularly those with poor plaque control and irregular maintenance during follow-up, exhibited an increased risk of developing peri-implantitis. In the context of orthodontic anchorage, poor OH may significantly increase the risk of peri-implantitis owing to the combined effects of bacterial biofilm accumulation, mechanical loading, and soft tissue stress, ultimately leading to accelerated MBL. Poor OH for implants used as orthodontic anchorage may accelerate tissue breakdown, especially in patients with a thin mucosal biotype, KM < 2 mm, and a history of periodontal disease, and deserves further evaluation.

The present study found no significant difference in the risk of peri-implantitis between orthodontic anchorage implants with KM < 2 mm and non-anchorage control implants. The need for KM around implants for maintaining tissue health and stability is debatable [38,39,40,41]. A KM < 2 mm around orthodontically loaded implants, especially with a history of periodontitis, significantly increases risks for peri-implantitis [38]. The impact of KM < 2 mm on implants used for orthodontic anchorage is unclear and may be associated with peri-implantitis owing to higher plaque accumulation, reduced patient comfort and hygiene, increased MBL, and soft tissue recession. Patients in our study were relatively highly compliant, closely monitored in a specialist setting, and received frequent maintenance. This limits external validity and should be acknowledged.

Several studies have evaluated the impact of loading forces on the performance of implant anchorage [42,43,44,45]. However, the optimal time interval and loading forces for dental implant stability sufficient for orthodontic movement remain debatable. The timing of implant loading as orthodontic anchorage was not significantly associated with peri-implantitis (Table 6). The effect of the loading timing on the performance of implant anchorages has been evaluated in several studies [46,47,48]. Immediate or early loading protocols, in which orthodontic forces are applied shortly after implant placement, can be effective if carefully managed. This approach reduces the treatment time and may enhance patient satisfaction. Delayed loading (>3 months) is considered safer for compromised sites. Careful planning, appropriate loading time and forces, high compliance, close monitoring in a specialist setting, and frequent SPC are warranted to avoid compromising the osseointegration for implant anchorage.

Although this retrospective long-term case–control study has some strengths, it also has certain limitations, such as recall and potential selection bias, incomplete data, small sample sizes, and uncontrolled confounding factors such as lack of standardized orthodontic and occlusal force calibration, implant diameter, implant length, bone quality, prosthetic design, loading time and absence of patient-reported outcomes. In this study, the small cohort size, combined with subgroup and risk modeling, undermines the statistical power and broad applicability. A non-significant result that might be important if the sample size was too small to detect a meaningful. Further prospective, long-term randomized controlled studies with larger sample sizes are warranted to validate these findings.

In the present study, each study group received a maximum of approximately one to two implants; therefore, the sample sizes at the implant and patient levels were not substantially different. Accordingly, results from most analyses were comparable between implant-level and patient-level approaches. Another limitation is sparse data bias in certain covariate strata. When events or covariate patterns are rare, penalized methods may yield unstable estimates with wide confidence intervals—particularly in subgroup analyses—potentially obscuring clinically meaningful associations [49].

## 5. Conclusions

The findings of this long-term case–control study suggest that none of the evaluated patient- or implant-related factors showed a statistically significant difference in the incidence of peri-implantitis between implants used as orthodontic anchorage and non-anchorage controls. Neither orthodontic anchorage nor implant type are significantly associated with MBL. However, poor patient compliance is a significant predictor of increased MBL. Poor OH and use of one-piece implants were moderately associated with a higher risk of peri-implantitis. Within the limitations of this study, dental implants were able to withstand both orthodontic and occlusal forces while maintaining long-term osseointegration in patients with Stage IV periodontitis.

## Figures and Tables

**Table 1:** Baseline characteristics of the patient- and implant-related factors for implants used as orthodontic anchorage and non-anchorage controls.

Variables	Non-Anchorage (Control) N (%)	Anchorage N (%)
*Patient-related Factors*	N = 29	N = 29
Age	49.44 ± 9.49	48.61 ± 9.24
Oral Hygiene		
Good	8 (27.6)	8 (27.6)
Medium	14 (48.3)	14 (48.3)
Poor	7 (24.1)	7 (24.1)
Compliance		
High	17 (58.6)	18 (62.1)
Medium	8 (27.6)	7 (24.1)
Low	4 (13.8)	4 (13.8)
*Implant-related Factors*	N = 29	N = 29
Implant position		
Maxilla	15 (51.7)	12 (41.4)
Mandible	14 (48.3)	17 (58.6)
Implant type		
Two-piece (Replace)	21 (72.4)	15 (51.7)
One-piece (Direct)	8 (27.6)	14 (48.3)
MBL > 1 mm (peri-implantitis)		
Yes	4 (13.8)	5 (17.2)
No	25 (86.2)	24 (82.8)
KM < 2 mm		
Yes	15 (51.7)	15 (51.7)
No	14 (48.3)	14 (48.3)
Tissue recession		
Recession (+)	8 (27.6)	10 (34.5)
Creeping (−)	3 (10.3)	1 (3.4)
None (0)	18 (62.1)	18 (62.1)
Follow-up years		
5–10	8 (27.6)	7 (24.1)
>10	21 (72.4)	22 (75.9)

Abbreviations: MBL, marginal bone loss; KM, keratinized mucosa.

**Table 2:** Changes in MBL for implants as orthodontic anchorage and implant type (N = 58).

	Changes in MBL			*p*-Value (*t*-Test)	*p*-Value (Wilcoxon)
Variables	Mean	±	SD		
Overall	0.35	±	1.02		
Orthodontic anchorage					
Yes	0.43	±	0.95	0.600	0.448
No	0.28	±	1.10		
Implant type					
Two-piece	0.18	±	0.64	0.089	0.210
One-piece	0.65	±	1.42		

Abbreviations: MBL, marginal bone loss; SD, standard deviation.

**Table 3:** Effects of patient- and implant-related factors on marginal bone loss of all implants (N = 58).

Variables	Coef.	95% CI	*p*-Value
*Patient-related Factors*			
Oral Hygiene			
Good (ref.)			
Medium	−0.07913	(−0.7112983, 0.5530334)	0.803
Poor	0.57552	(−0.1626936, 1.3137277)	0.124
Compliance			
High (ref.)			
Medium	−0.07767	(−0.6725080, 0.5171587)	0.794
Low	1.11945	(0.3641047, 1.8747941)	0.004
*Implant-related Factors*			
Implant type			
Two-piece (ref.)			
One-piece	0.4709	(−0.07565751, 1.0174195)	0.089
KM < 2 mm			
No (ref.)			
Yes	0.50873	(−0.0185896, 1.0360579)	0.058
Tissue recession			
None (ref.) (0)			
Recession (+)	0.2571	(−0.34059684, 0.8547723)	0.392
Creeping (−)	−0.2616	(−1.35286026, 0.8295753)	0.632
Follow-up years			
<10 (ref.)			
>10	0.1769	(−0.4428270, 0.7966073)	0.570

Abbreviations: Coef, coefficient; CI, confidence interval; KM, keratinized mucosa.

**Table 4:** Baseline characteristic distribution stratified by peri-implantitis (N = 58).

Variables	Changed MBL ≤1 mm (n = 49)	Changed MBL >1 mm (n = 9)	*p*-Value
*Patient-related Factors*			
Oral Hygiene			0.052
Good	15 (30.6%)	1 (11.1%)	
Medium	25 (51%)	3 (33.3%)	
Poor	9 (18.4%)	5 (55.6%)	
Compliance			0.143
High	30 (61.2%)	5 (55.6%)	
Medium	14 (28.6%)	1 (11.1%)	
Low	5 (10.2%)	3 (33.3%)	
*Implant-related Factors*			
Implant type.			0.053
Two-piece	16 (32.7%)	6 (66.7%)	
One-piece	33 (67.3%)	3 (33.3%)	
KM < 2 mm			
No	2 (8.4%)	1 (11.1%)	0.999
Yes	22 (92.6%)	8 (80.9%)	
Tissue recession			0.169
Recession (+)	14 (28.6%)	4 (44.4%)	
Creeping (−)	3 (6.1%)	1 (11.1%)	
None (0)	32 (65.3%)	4 (44.4%)	
Follow-up years			0.330
Mean ± SD	11.74 ± 3.87	13.05 ± 2.17	

Abbreviations: MBL, marginal bone loss; KM, keratinized mucosa.

**Table 5:** Effects of patient- and implant-related factors on peri-implantitis for implants used as orthodontic anchorage and non-anchorage controls.

Variables	Non-Anchorage MBL ≤ 1 mm n = 25 (%)	Non-Anchorage MBL > 1 mm n = 4 (%)	Anchorage MBL ≤ 1 mm n = 24 (%)	Anchorage MBL > 1 mm n = 5 (%)	*p*-Value
*Patient-related Factors*					
Oral Hygiene					
Good	8 (32)	0 (0)	7 (29.1)	1 (20)	0.328
Medium	12 (48)	2 (50)	13 (54.2)	1 (20)	
Poor	5 (20)	2 (50)	4 (16.7)	3 (60)	
Compliance					
High	14 (56)	3 (75)	16 (66.7)	2 (40)	0.466
Medium	8 (32)	0 (0)	6 (25)	1 (20)	
Low	3 (12)	1 (25)	2 (8.3)	2 (40)	
*Implant-related Factors*					
Implant position					
Maxilla	13 (52)	2 (50)	10 (41.7)	2 (40)	0.888
Mandible	12 (48)	2 (50)	14 (58.3)	3 (60)	
Implant type					
Two-piece	19 (76)	2 (50)	14 (58.3)	1 (20)	0.101
One-piece	6 (24)	2 (50)	10 (41.7)	4 (80)	
KM < 2 mm					
No	13 (52)	1 (25)	14 (58.3)	0 (0)	0.084
Yes	12 (48)	3 (75)	10 (41.7)	5 (100)	
Tissue recession					
Recession (+)	6 (24)	2 (50)	8 (33.3)	2 (40)	0.391
Creeping (−)	3 (12)	0 (0)	0	1 (20)	
None (0)	16 (64)	2 (50)	16 (66.7)	2 (40)	
Follow-up years					
Mean ± SD	11.50 ± 3.89	12.25 ± 2.39	12.00 ± 3.91	13.70 ± 1.98	0.684

Abbreviations: MBL, marginal bone loss; KM, keratinized mucosa.

**Table 6:** Impact of orthodontic loading time on peri-implantitis (N = 9).

	Changes in MBL > 1 mm			*p*-Value
Variables	Mean	±	SD	
Overall	2.11	±	1.37	
>3 M (ref.)	1.94	±	0.44	0.741
1–3 M	2.34	±	1.64	
<1 M	1.08	±	-	

Abbreviations: MBL, marginal bone loss; SD, standard deviation.

## Data Availability

The data that support the findings of this study are available on request from the corresponding author. The data are not publicly available due to privacy or ethical restrictions.

## Supplementary Materials

The following supporting information can be downloaded at: https://www.mdpi.com/article/10.3390/jfb17010049/s1, Table S1. STROBE Statement—checklist of items that should be included in reports of observational studies.

## Abbreviations

BOP	Bleeding on probing
GEE	Generalized estimating equation
KM	Keratinized mucosa
MBCP	Macroporous biphasic calcium phosphate
MBL	Marginal bone level
MTR	Midfacial mucosal tissue recession
OH	Oral Hygiene
PS	Plaque score
SPC	Supportive periodontal and peri-implant care
